# Optical soliton solutions of the coupled Radhakrishnan-Kundu-Lakshmanan equation by using the extended direct algebraic approach

**DOI:** 10.1016/j.heliyon.2023.e20852

**Published:** 2023-10-13

**Authors:** Ayesha Mahmood, Hari Mohan Srivastava, Muhammad Abbas, Farah Aini Abdullah, Pshtiwan Othman Mohammed, Dumitru Baleanu, Nejmeddine Chorfi

**Affiliations:** a Department of Mathematics, University of Sargodha, Sargodha 40100, Pakistan; b Department of Mathematics and Statistics, University of Victoria, Victoria, British Columbia V8W 3R4, Canada; c Center for Converging Humanities, Kyung Hee University, 26 Kyungheedae-ro, Dongdaemun-gu, Seoul 02447, Republic of Korea; d Section of Mathematics, International Telematic University Uninettuno, I-00186 Rome, Italy; e School of Mathematical Sciences, Universiti Sains Malaysia, 11800 Penang, Malaysia; f Department of Mathematics, College of Education, University of Sulaimani, Sulaimani 46001, Kurdistan Region, Iraq; g Department of Computer Science and Mathematics, Lebanese American University, Beirut 11022801, Lebanon; h Institute of Space Sciences, R76900 Magurele-Bucharest, Romania; i Department of Medical Research, China Medical University, Taichung 40402, Taiwan; j Department of Mathematics, College of Science, King Saud University, P.O. Box 2455, Riyadh 11451, Saudi Arabia

**Keywords:** 39A12, 39B62, 33B10, 26A48, 26A51, Extended direct algebraic (EDA) technique, Radhakrishnan-Kundu-Lakshmanan equation (RKLE), Optical solitons, Soliton solutions, Birefringent fibres

## Abstract

The analytical soliton solutions place a lot of value on birefringent fibres. The major goal of this study is to generate novel forms of soliton solutions for the Radhakrishnan-Kundu-Lakshmanan equation, which depicts unstable optical solitons that arise from optical propagations using birefringent fibres. The (presumably new) extended direct algebraic (EDA) technique is used here to extract a large number of solutions for RKLE. It gives soliton solutions up to thirty-seven, which essentially correspond to all soliton families. This method's ability to determine many sorts of solutions through a single process is one of its key advantages. Additionally, it is simple to infer that the technique employed in this study is really straightforward yet one of the quite effective approaches to solving nonlinear partial differential equations so, this novel extended direct algebraic (EDA) technique may be regarded as a comprehensive procedure. The resulting solutions are found to be hyperbolic, periodic, trigonometric, bright and dark, combined bright-dark, and W-shaped soliton, and these solutions are visually represented by means of 2D, 3D, and density plots. The present study can be extended to investigate several other nonlinear systems to understand the physical insights of the optical propagations through birefringent fibre.

## 1 Introduction

Nonlinear partial differential equations (PDEs) are important for modelling, analyzing and explaining a wide range of scientific phenomena. It is necessary to solve the models under discussion precisely by using suitable methods. The study of exact solutions to nonlinear PDEs has been an active field of research which plays an important role in the study of their applications in the real world [1], [2], [3], [4]. Various computational techniques have been proposed until now for obtaining the exact solutions to nonlinear equations. For instance, to determine solitary, periodic, and compacton-like solutions, the exp-function approach is utilized [5], a number of exact solutions for travelling waves may be produced by using the extended trial equation approach [6], the voltage in transmission line issues can be observed by using different methods [7], and it is also possible to build some non-topological solitons and closed-form solutions [8]. The phenomenon of travelling waves is widely investigated by means of the soliton theory which is an effective area of study for nonlinear partial differential equations like Kodomtsev–Petviashvili (KP) equation, the Schrodinger equation, Kundu-Eckhaus equation and many more that arise in disciplines like mathematical physics, nuclear physics, optics, and telecom engineering [9], [10], [11]. As a result, the researchers have created a wide range of analytical schemes to develop different type of solutions like the rational, rogue, breather, solitary, periodic, singular and optical wave solutions [12]. Several analytical methods have been employed to create the soliton solutions, including the modified auxiliary equation method [13], the Jacobi elliptic function method [14], the sine-Gordon expansion method [15], and the Lie symmetry analysis [16]. One of the nature's most fascinating nonlinear wave occurrences is the soliton, which is defined as a self-sustaining localized structure [17], [18], [19], [20]. By simulating the occurrence in a wave tank, John Scott Russell initially proposed the concept of solitons in 1834. Optical solitons are a particular kind of solitary waves that travel over great distances without dispersing. They were found by Zhakarov and Sabat in 1971. In optical theory, linear and nonlinear effects are weakly balanced in the medium, so solitons may be used to refer to any optical field that does not vary throughout propagation. Optical solitons have been divided into spatial and temporal solitons. The dynamics of optical solitons are essential to the growth of the telecommunication sector. Due to their wide applicability the study of optical solitons solutions are considered by professionals and academics, in recent years. Numerous significant methods have been put out, including motion of solitary waves in the nonlinear optics [21], lipid sensor [22], the analysis method [23], generalized Jacobi elliptic expansion method [24], the trial equation technique [25], propagation of ultrashort optical pulses [26], pulses propagation in nonlinear optical systems [27], modulation instability criteria [28], stability of solitons [29], evolution of dark solitons [30], intermodal dispersion effect [31], and soliton solutions in birefringent optical fibre [32]. Soliton propagation dynamics through a fibre with maintained polarization are governed by the Radhakrishnan-Kundu-Lakshmanan equation (RKLE) which is a generalized version of the nonlinear Schrodinger's equation. Radhakrishnan, Kundu, and Lakshmanan initially introduced RKLE in 1976, and it has since been used in a wide variety of physics, chemistry, and engineering contexts. The Radhakrishnan-Kundu-Lakshmanan equation (RKLE) has been used widely by recent researchers in order to examine how optical solitons move along optical fibres [33], [34], [35], [36]. The Radhakrishnan-Kundu-Lakshmanan equation (RKLE) may be used to study dispersive optical solitons exhibiting the Kerr law nonlinearity without the four-wave mixing (4WM) effect which is regarded as being the basic case of fibre nonlinearity. The recent popularity of several optical fibres makes them compliant with this law. This medium shows self-phase modulation, and the frequency-shifting of a pulse of light as it moves towards a fibre nonlinearity [37]. Consequently, in this work, we investigate the coupled Radhakrishnan-Kundu-Lakshmanan equation to obtain the soliton solutions by using a (presumably new) extended direct algebraic (EDA) technique. In its dimensionless form, RKLE is given as follows (see [38], [39], [40]):

(1.1) $$\iota U_{t}+dU_{rr}+e|U{|}^{2}U=\iota \mu {\left(|U{|}^{2}U\right)}_{r}-\iota \nu U_{rrr}\;(\iota :=\sqrt{-1}),$$

where *U* is a wave profile having complex values, *d* is the chromatic dispersion coefficient, *e* is the Kerr nonlinearity, *μ* is the self-steepening coefficient, and *ν* is the third-order dispersion coefficient.

The coupled Radhakrishnan-Kundu-Lakshmanan equation (RKLE) is a nonlinear PDE that explains how coupled oscillations behave dynamically in a system of two or more oscillatory components. It can be regarded as given below (see [41], [42]):

(1.2) $$\iota U_{t}+d_{1}U_{rr}+(e_{1}|U{|}^{2}+f_{1}|V{|}^{2})U=\iota (\mu_{1}{\left(|U{|}^{2}U\right)}_{r}+\nu_{1}U_{r})-\iota \omega_{1}U_{rrr},\iota V_{t}+d_{2}V_{rr}+(e_{2}|V{|}^{2}+f_{2}|U{|}^{2})V=\iota (\mu_{2}{\left(|V{|}^{2}V\right)}_{r}+\nu_{2}V_{r})-\iota \omega_{2}V_{rrr}.$$

The wave profiles are presented by the wave potentials U(r,t) and V(r,t). These wave potentials take on complex values. Here, for i=1,2, $e_{i}$ is the self-phase modulation, $f_{i}$ shows the cross-phase modulation, $\mu_{i}$ and $\nu_{i}$ are the self-steepings when there is no four-wave mixing (4WM).

In this paper, the novel extended direct algebraic approach is used to provide a successful explanation for the dynamical behaviour of optical solitons of the RKLE without four wave mixing (4WM) effect.

The article is structured as follows: The mathematical computation for the coupled Radhakrishnan-Kundu-Lakshmanan equation (RKLE) is explained in Section 2. Explanation of the EDA approach is given in Section 3. Section 4 covers the application of the EDA technique to the RKLE and the graphical description is presented in Section 5. Finally, conclusion is given in Section 6.

## 2 Mathematical computation

Let us suppose the following transformations for both *U* and *V*, respectively.

(2.1) $$U(r,t)=e^{\iota \xi (r,t)}\chi_{1}(\eta ),V(r,t)=e^{\iota \xi (r,t)}\chi_{2}(\eta ),$$

where $\xi (r,t)=-ar+bt+\theta_{0}$ and η=r−ct. Furthermore, $\chi_{i}(\eta )$ gives the amplitude of soliton i=(1,2), *c* is the speed of wave and *a* is the frequency, whereas *b* represents the wave number, ξ(r,t) is phase component of pulse and $\theta_{0}$ is the phase constant.

Putting Equation (2.1) with the transformations in Equation (1.2) and separating the real and imaginary parts, we get

(2.2) $$-d_{i}a^{2}\chi_{i}-a^{3}\omega_{i}\chi_{i}-b\chi_{i}+e_{i}\chi_{i}^{3}+f_{i}\chi_{i}\chi_{j}^{2}-a\mu_{i}\chi_{j}^{3}-a\nu_{i}\chi_{i}\chi_{j}^{2}+d_{i}{\chi_{i}}^{\prime \prime }+3a\omega_{i}{\chi_{i}}^{\prime \prime }=0,$$

and

(2.3) $$-2d_{i}a{\chi_{i}}^{\prime }-d{\chi_{i}}^{\prime }-3a^{2}\omega_{i}{\chi_{i}}^{\prime }-3\mu_{i}\chi_{i}^{2}{\chi_{i}}^{\prime }-\nu_{i}\chi_{j}^{2}{\chi_{j}}^{\prime }-2\nu_{i}\chi_{i}\chi_{j}{\chi_{j}}^{\prime }+\omega_{i}{\chi_{i}}^{\prime \prime \prime }=0.$$

Equation (2.2) and Equation (2.3) imply that $\chi_{i}=\chi_{j}$ by using the balancing principle for both i=1,2 and j=3−i.

(2.4) $$-d_{i}a^{2}\chi_{i}-a^{3}\omega_{i}\chi_{i}-b\chi_{i}+e_{i}\chi_{i}^{3}+f_{i}\chi_{i}^{3}-a\mu_{i}\chi_{i}^{3}-a\nu_{i}\chi_{i}^{3}+d_{i}{\chi_{i}}^{\prime \prime }+3a\omega_{i}{\chi_{i}}^{\prime \prime }=0,$$

and

(2.5) $$-2d_{i}a{\chi_{i}}^{\prime \prime }-d{\chi_{i}}^{\prime }-3a^{2}\omega_{i}{\chi_{i}}^{\prime }-3\mu_{i}\chi_{i}^{2}{\chi_{i}}^{\prime }-\nu_{i}\chi_{i}^{2}{\chi_{i}}^{\prime }-2\nu_{i}\chi_{i}^{2}{\chi_{i}}^{\prime }+\omega_{i}{\chi_{i}}^{\prime \prime \prime }=0.$$

Integrating Equation (2.5) with respect to *η* and putting the constant of integration equal to zero, we get

(2.6) $$-(2d_{i}a+c+3a^{2}\nu_{i})\chi_{i}-(\mu_{i}+\nu_{i})\chi_{i}^{3}+\omega_{i}{\chi_{i}}^{\prime \prime }=0.$$

Equation (2.4) and Equation (2.6) are the same if and only if

(2.7) $$\frac{d_{i}+3a\omega_{i}}{\omega_{i}}=\frac{-(d_{i}a^{2}+a^{3}\omega_{i}+b)}{2d_{i}a+c+3a^{2}\omega_{i}}=\frac{e_{i}+f_{i}-a\mu_{i}-a\nu_{i}}{-(\mu_{i}+\nu_{i})},$$

where

$$e_{i}=\frac{f_{i}\omega_{i}+d_{i}\mu_{i}+2a\omega_{i}\mu_{i}+d_{i}\nu_{i}+2a\omega_{i}\nu_{i}}{\omega_{i}},$$

and

$$b=\frac{2d_{i}^{2}a+d_{i}c+8d_{i}a^{2}\omega_{i}+3ac\omega_{i}+8a^{3}\omega_{i}^{2}}{\omega_{i}}.$$

## 3 Explanation of the EDA technique

Let us consider a general nonlinear PDE given by

(3.1) $$G(\chi ,\chi_{t},\chi_{r},\chi_{tt},\chi_{rr},\chi_{rt},\cdots )=0,$$

which can be converted into an ODE of the following form:

(3.2) $$H(Y,Y^{\prime },Y^{\prime \prime },\cdots )=0,$$

by using the following transformation:

(3.3) $$\chi (r,t)=Y(\xi )e^{\iota \eta },$$

where $\xi =m_{1}r+m_{2}t$ and $\eta =m_{3}r+m_{4}t$.

We assume that Equation (3.2) has a solution of the following form:

(3.4) $$Y(\xi )=\sum_{k=0}^{N}h_{k}F^{k}(\xi ),$$

where

(3.5) $$F^{\prime }(\xi )=\ln\tau \left(\theta +\phi F(\xi )+\psi {\left(F(\xi )\right)}^{2}\right)\;(\tau \neq 0,1),$$

and *θ*, *ϕ* and *ψ* are constants having real values. The general form of the solutions of Equation (3.5) with respect to these parameters is given as detailed below:

**1.** For $\phi^{2}-4\theta \psi <0$ and ψ≠0, we have

(3.6) $$F_{1}(\xi )=-\frac{\phi }{2\psi }+\frac{\sqrt{-\rho }}{2\psi }\tan_{\tau }\left(\frac{\sqrt{-\rho }}{2}\xi \right),$$

(3.7) $$F_{2}(\xi )=-\frac{\phi }{2\psi }-\frac{\sqrt{-\rho }}{2\psi }\cot_{\tau }\left(\frac{\sqrt{-\rho }}{2}\xi \right),$$

(3.8) $$F_{3}(\xi )=-\frac{\phi }{2\psi }+\frac{\sqrt{-\rho }}{2\psi }\left(\tan_{\tau }\left(\sqrt{-\rho }\xi \right)\pm \sqrt{\delta \sigma }\sec_{\tau }\left(\sqrt{-\rho }\xi \right)\right),$$

(3.9) $$F_{4}(\xi )=-\frac{\phi }{2\psi }+\frac{\sqrt{-\rho }}{2\psi }\left(\cot_{\tau }\left(\sqrt{-\rho }\xi \right)\pm \sqrt{\delta \sigma }\csc_{\tau }\left(\sqrt{-\rho }\xi \right)\right)$$

and

(3.10) $$F_{5}(\xi )=-\frac{\phi }{2\psi }+\frac{\sqrt{-\rho }}{4\psi }\left(\tan_{\tau }\left(\frac{\sqrt{-\rho }}{4}\xi \right)-\cot_{\tau }\left(\frac{\sqrt{-\rho }}{4}\xi \right)\right).$$

**2.** For $\phi^{2}-4\theta \psi >0$ and ψ≠0, we have

(3.11) $$F_{6}(\xi )=-\frac{\phi }{2\psi }-\frac{\sqrt{\rho }}{2\psi }\tanh_{\tau }\left(\frac{\sqrt{\rho }}{2}\xi \right),$$

(3.12) $$F_{7}(\xi )=-\frac{\phi }{2\psi }-\frac{\sqrt{\rho }}{2\psi }\coth_{\tau }\left(\frac{\sqrt{\rho }}{2}\xi \right),$$

(3.13) $$F_{8}(\xi )=-\frac{\phi }{2\psi }+\frac{\sqrt{\rho }}{2\psi }\left(-\tanh_{\tau }\left(\sqrt{\rho }\xi \right)\pm \iota {\sqrt{\delta \sigma }}_{\tau }\left(\sqrt{\rho }\xi \right)\right),$$

(3.14) $$F_{9}(\xi )=-\frac{\phi }{2\psi }+\frac{\sqrt{\rho }}{2\psi }\left(-\coth_{\tau }\left(\sqrt{\rho }\xi \right)\pm {\sqrt{\delta \sigma }}_{\tau }\left(\sqrt{\rho }\xi \right)\right)$$

and

(3.15) $$F_{10}(\xi )=-\frac{\phi }{2\psi }-\frac{\sqrt{\rho }}{4\psi }\left(\tanh_{\tau }\left(\frac{\sqrt{\rho }}{4}\xi \right)+\coth_{\tau }\left(\frac{\sqrt{\rho }}{4}\xi \right)\right).$$

**3.** For θψ>0 and ϕ=0, we have

(3.16) $$F_{11}(\xi )=\sqrt{\frac{\theta }{\psi }}\tan_{\tau }\left(\sqrt{\theta \psi }\xi \right),$$

(3.17) $$F_{12}(\xi )=-\sqrt{\frac{\theta }{\psi }}\cot_{\tau }\left(\sqrt{\theta \psi }\xi \right),$$

(3.18) $$F_{13}(\xi )=\sqrt{\frac{\theta }{\psi }}\left(\tan_{\tau }\left(2\sqrt{\theta \psi }\xi \right)\pm \sqrt{\delta \sigma }\sec_{\tau }\left(2\sqrt{\theta \psi }\xi \right)\right),$$

(3.19) $$F_{14}(\xi )=\sqrt{\frac{\theta }{\psi }}\left(-\cot_{\tau }\left(2\sqrt{\theta \psi }\xi \right)\pm \sqrt{\sigma }\csc_{\tau }\left(2\sqrt{\theta \psi }\xi \right)\right)$$

and

(3.20) $$F_{15}(\xi )=\frac{1}{2}\sqrt{\frac{\theta }{\psi }}\left(\tan_{\tau }\left(\frac{\sqrt{\theta \psi }}{2}\xi \right)+\cot_{\tau }\left(\frac{\sqrt{\theta \psi }}{2}\xi \right)\right).$$

**4.** For θψ<0 and ϕ=0, we have

(3.21) $$F_{16}(\xi )=-\sqrt{-\frac{\theta }{\psi }}\tanh_{\tau }\left(\sqrt{-\theta \psi }\xi \right),$$

(3.22) $$F_{17}(\xi )=-\sqrt{-\frac{\theta }{\psi }}\coth_{\tau }\left(\sqrt{-\theta \psi }\xi \right),$$

(3.23) $$F_{18}(\xi )=\sqrt{-\frac{\theta }{\psi }}\left(-\tanh_{\tau }\left(2\sqrt{-\theta \psi }\xi \right)\pm \iota {\sqrt{\delta \sigma }}_{\tau }\left(2\sqrt{-\theta \psi }\xi \right)\right),$$

(3.24) $$F_{19}(\xi )=\sqrt{-\frac{\theta }{\psi }}\left(-\coth_{\tau }\left(2\sqrt{-\theta \psi }\xi \right)\pm {\sqrt{\delta \sigma }}_{\tau }\left(2\sqrt{-\theta \psi }\xi \right)\right)$$

and

(3.25) $$F_{20}(\xi )=-\frac{1}{2}\sqrt{-\frac{\theta }{\psi }}\left(\tanh_{\tau }\left(\frac{\sqrt{-\theta \psi }}{2}\xi \right)+\coth_{\tau }\left(\frac{\sqrt{-\theta \psi }}{2}\xi \right)\right).$$

**5.** For ϕ=0 and θ=ψ, we have

(3.26) $$F_{21}(\xi )=\tan_{\tau }\left(\theta \xi \right)$$

(3.27) $$F_{22}(\xi )=-\cot_{\tau }\left(\theta \xi \right)$$

(3.28) $$F_{23}(\xi )=\tan_{\tau }\left(2\theta \xi \right)\pm \sqrt{\delta \sigma }\sec_{\tau }\left(2\theta \xi \right),$$

(3.29) $$F_{24}(\xi )=-\cot_{\tau }\left(2\theta \xi \right)\pm \sqrt{\delta \sigma }\csc_{\tau }\left(2\theta \xi \right)$$

and

(3.30) $$P_{25}(\xi )=\frac{1}{2}\left(\tan_{\tau }\left(\frac{\theta }{2}\xi \right)-\cot_{\tau }\left(\frac{\theta }{2}\xi \right)\right).$$

**6.** For ϕ=0 and ψ=−θ, we have

(3.31) $$F_{26}(\xi )=-\tanh_{\tau }\left(\theta \xi \right),$$

(3.32) $$F_{27}(\xi )=-\coth_{\tau }\left(\theta \xi \right),$$

(3.33) $$F_{28}(\xi )=-\tanh_{\tau }\left(2\theta \xi \right)\pm \iota {\sqrt{\delta \sigma }}_{\xi }\left(2\theta \xi \right),$$

(3.34) $$F_{29}(\xi )=-\coth_{\tau }\left(2\theta \xi \right)\pm {\sqrt{\delta \sigma }}_{\tau }\left(2\theta \xi \right)$$

and

(3.35) $$F_{30}(\xi )=-\frac{1}{2}\left(\tanh_{\tau }\left(\frac{\theta }{2}\xi \right)+\coth_{\tau }\left(\frac{\theta }{2}\xi \right)\right).$$

**7.** For $\phi^{2}=4\theta \psi$, we have

(3.36) $$F_{31}(\xi )=\frac{-2\theta (\phi \xi \ln(\tau )+2)}{\phi^{2}\xi \ln(\tau )}.$$

**8.** For ϕ=x,θ=xy(y≠0) and ψ=0, we have

(3.37) $$F_{32}(\xi )=\tau^{x\xi }-y.$$

**9.** For θ=ψ=0, we have

(3.38) $$F_{33}(\xi )=\theta \xi \ln(\tau ).$$

**10.** For ϕ=θ=0, we have

(3.39) $$F_{34}(\xi )=\frac{-1}{\psi \xi \ln(\tau )}.$$

**11.** For ϕ≠0 and θ=0, we have

(3.40) $$F_{35}(\xi )=-\frac{\delta \phi }{\psi \left(\cosh_{\tau }(\phi \xi )-\sinh_{\tau }(\phi \xi )+\delta \right)}$$

and

(3.41) $$F_{36}(\xi )=-\frac{\phi \left(\sinh_{\tau }(\phi \xi )+\cosh_{\tau }(\phi \xi )\right)}{\psi \left(\sinh_{\tau }(\phi \xi )+\cosh_{\tau }(\phi \xi )+\sigma \right)}.$$

**12.** For ϕ=x,ψ=xy(y≠0) and θ=0, we have

(3.42) $$F_{37}(\xi )=-\frac{m\tau^{x\xi }}{\delta -y\sigma \tau^{x\xi }},$$

(3.43) $$\sinh_{\tau }(\xi )=\frac{\delta \tau^{\xi }-\sigma \tau^{-\xi }}{2},\cosh_{\tau }(\xi )=\frac{\delta \tau^{\xi }+\sigma \tau^{-\xi }}{2},\tanh_{\tau }(\xi )=\frac{\delta \tau^{\xi }-\sigma \tau^{-\xi }}{\delta \tau^{\xi }+\sigma \tau^{-\xi }},$$

(3.44) $$\csc_{\tau }(\xi )=\frac{2}{\delta \tau^{\xi }-\sigma \tau^{-\xi }},\sec_{\tau }(\xi )=\frac{2}{\delta \tau^{\xi }+\sigma \tau^{-\xi }},\coth_{\tau }(\xi )=\frac{\delta \tau^{\xi }+\sigma \tau^{-\xi }}{\delta \tau^{\xi }-\sigma \tau^{-\xi }},$$

(3.45) $$\sin_{\tau }(\xi )=\frac{\delta \tau^{\iota \xi }-\sigma \tau^{-\iota \xi }}{2\iota },\cos_{\tau }(\xi )=\frac{\delta \tau^{\iota \xi }+\sigma \tau^{-\iota \xi }}{2},\tan_{\tau }(\xi )=-\iota \frac{\delta \tau^{\iota \xi }-\sigma \tau^{-\iota \xi }}{\delta \tau^{\iota \xi }+\sigma \tau^{-\iota \xi }}$$

and

(3.46) $$\csc_{\tau }(\xi )=\frac{2\iota }{\delta \tau^{\xi }-\sigma \tau^{-\xi }},\sec_{\tau }(\xi )=\frac{2}{\delta \tau^{\xi }+\sigma \tau^{-\xi }},\cot_{\rho }(\xi )=\iota \frac{\delta \tau^{\iota \xi }+\sigma \tau^{-\iota \xi }}{\delta \tau^{\iota \xi }-\sigma \tau^{-\iota \xi }},$$

where the deformation parameters δ>0 and σ>0 are arbitrary constants.

## 4 Application involving RKLE

By using the transformations, we get Equation (2.5). Therefore, the homogeneous balancing constant N=1 between ${\chi_{i}}^{\prime \prime }$ and $\chi_{i}^{3}$. Thus, the solution is given as follows:

(4.1) $$Y_{i}(\xi )=h_{0}+h_{1}F(\xi ),$$

where

(4.2) $$F^{\prime }(\xi )=\ln(\tau )\left(\theta +\phi F+\psi {\left(F(\xi )\right)}^{2}\right).$$

Upon substituting the solution of Equation (4.1) and Equation (4.2), we collect the coefficients of powers of F(ξ). Thus, an algebraic system of equations is obtained. Solving that system, we get

(4.3) $$h_{0}=\Delta \phi ,h_{1}=2\Delta \psi ,$$

where

$$\Delta =-\iota ln(\tau )\sqrt{\frac{H_{i,0}}{2H_{i,1}}}.$$

We also have

$$H_{i,0}=1,\;H_{i,1}=\frac{-(\mu_{i}+\nu_{i})}{\omega_{i}}\;\text{and}\;H_{i,2}=\frac{-(2d_{i}a+c+3a^{2}\nu_{i})}{\omega_{i}}.$$

The following general solution of Equation (1.2) is obtained by substituting Equation (4.3) into Equation (4.1):

(4.4) $$Y_{i}(r,t)=\Delta \phi +2\Delta \psi F_{g}(\xi ).$$

Here, we have $\rho =\phi^{2}-4\theta \psi$. After using different values of $F_{g}$ from Equation (3.6) to (3.42), we get many corresponding solutions.

(1) For $\phi^{2}-4\theta \psi <0$ and ψ≠0, we have

(4.5) $$\chi_{i,1}(r,t)=\left[\Delta \sqrt{-\rho }\tan_{\tau }\left(\frac{\sqrt{-\rho }}{2}\xi \right)\right]e^{\iota \eta },$$

(4.6) $$\chi_{i,2}(r,t)=-\left[\Delta \sqrt{-\rho }\cot_{\tau }\left(\frac{\sqrt{-\rho }}{2}\xi \right)\right]e^{\iota \eta },$$

(4.7) $$\chi_{i,3}(r,t)=\left[\Delta \sqrt{-\rho }\left(\tan_{\tau }\left(\sqrt{-\rho }\xi \right)\pm \sqrt{\delta \sigma }\sec_{\tau }\left(\sqrt{-\rho }\xi \right)\right)\right]e^{\iota \eta },$$

(4.8) $$\chi_{i,4}(r,t)=\left[\Delta \sqrt{-\rho }\left(\cot_{\tau }\left(\sqrt{-\rho }\xi \right)\pm \sqrt{\delta \sigma }\csc_{\tau }\left(\sqrt{-\rho }\xi \right)\right)\right]e^{\iota \eta }$$

and

(4.9) $$\chi_{i,5}(r,t)=\left[\Delta \frac{\sqrt{-\rho }}{4}\left(\tan_{\tau }\left(\frac{\sqrt{-\rho }}{4}\xi \right)-\cot_{\tau }\left(\frac{\sqrt{-\rho }}{4}\xi \right)\right)\right]e^{\iota \eta },$$

**(2)** For $\phi^{2}-4\theta \psi >0$ and ψ≠0, we have

(4.10) $$\chi_{i,6}(r,t)=-\left[\Delta \sqrt{\rho }\tanh_{\tau }\left(\frac{\sqrt{\rho }}{2}\xi \right)\right]e^{\iota \eta },$$

(4.11) $$\chi_{i,7}(r,t)=-\left[\Delta \sqrt{\rho }\coth_{\tau }\left(\frac{\sqrt{\rho }}{2}\xi \right)\right]e^{\iota \eta },$$

(4.12) $$\chi_{i,8}(r,t)=\left[\Delta \sqrt{\rho }\left(-\tanh_{\tau }\left(\sqrt{\rho }\xi \right)\pm \iota {\sqrt{\delta \sigma }}_{\tau }\left(\sqrt{\rho }\xi \right)\right)\right]e^{\iota \eta },$$

(4.13) $$\chi_{i,9}(r,t)=\left[\Delta \sqrt{\rho }\left(-\coth_{\tau }\left(\sqrt{\rho }\xi \right)\pm {\sqrt{\delta \sigma }}_{\tau }\left(\sqrt{\rho }\xi \right)\right)\right]e^{\iota \eta }$$

and

(4.14) $$\chi_{i,10}(r,t)=\left[\frac{\Delta }{2}\sqrt{\rho }\left(\tanh_{\tau }\left(\frac{\sqrt{\rho }}{4}\xi \right)+\coth_{\tau }\left(\frac{\sqrt{\rho }}{4}\xi \right)\right)\right]e^{\iota \eta }.$$

**(3)** For θψ>0 and ϕ=0, we have

(4.15) $$\chi_{i,11}(r,t)=\left[2\Delta \sqrt{\theta \psi }\left(\tan_{\tau }\left(\sqrt{\theta \psi }\xi \right)\right)\right]e^{\iota \eta },$$

(4.16) $$\chi_{i,12}(r,t)=-\left[2\Delta \sqrt{\theta \psi }\left(\cot_{\tau }\left(\sqrt{\theta \psi }\xi \right)\right)\right]e^{\iota \eta },$$

(4.17) $$\chi_{i,13}(r,t)=\left[2\Delta \sqrt{\theta \psi }\left(\tan_{\tau }\left(2\sqrt{\theta \psi }\xi \right)\right)\pm \sqrt{\delta \sigma }\sec_{\tau }\left(2\sqrt{\theta \psi }\xi \right)\right]e^{\iota \eta },$$

(4.18) $$\chi_{i,14}(r,t)=\left[2\Delta \sqrt{\theta \psi }\left(-\cot_{\tau }\left(2\sqrt{\theta \psi }\xi \right)\right)\pm \sqrt{\delta \sigma }\csc_{\tau }\left(2\sqrt{\theta \psi }\xi \right)\right]e^{\iota \eta }$$

and

(4.19) $$\chi_{i,15}(r,t)=\left[\Delta \sqrt{\theta \psi }\left(\tan_{\tau }\left(\frac{\sqrt{\theta \psi }}{2}\xi \right)-\cot_{\tau }\left(\frac{\sqrt{\theta \psi }}{2}\xi \right)\right)\right]e^{\iota \eta }.$$

**(4)** For θψ<0 and ϕ=0, we have

(4.20) $$\chi_{i,16}(r,t)=-\left[2\Delta \sqrt{-\theta \psi }\left(\tanh_{\tau }\left(\sqrt{-\theta \psi }\xi \right)\right)\right]e^{\iota \eta },$$

(4.21) $$\chi_{i,17}(r,t)=-\left[2\Delta \sqrt{-\theta \psi }\left(\coth_{\tau }\left(\sqrt{-\theta \psi }\xi \right)\right)\right]e^{\iota \eta },$$

(4.22) $$\chi_{i,18}(r,t)=\left[2\Delta \sqrt{-\theta \psi }\left(-\tanh_{\tau }\left(2\sqrt{-\theta \psi }\xi \right)\right)\pm \iota {\sqrt{\delta \sigma }}_{\tau }\left(2\sqrt{-\theta \psi }\xi \right)\right]e^{\iota \eta },$$

(4.23) $$\chi_{i,19}(r,t)=\left[2\Delta \sqrt{-\theta \psi }\left(-\coth_{\tau }\left(2\sqrt{-\theta \psi }\xi \right)\right)\pm {\sqrt{\delta \sigma }}_{\tau }\left(2\sqrt{-\theta \psi }\xi \right)\right]e^{\iota \eta }$$

and

(4.24) $$\chi_{i,20}(r,t)=-\left[\Delta \sqrt{-\theta \psi }\left(\tanh_{\tau }\left(\frac{\sqrt{-\theta \psi }}{2}\xi \right)+\coth_{\tau }\left(\frac{\sqrt{-\theta \psi }}{2}\xi \right)\right)\right]e^{\iota \eta }.$$

**(5)** For ϕ=0 and θ=ψ, we have

(4.25) $$\chi_{i,21}(r,t)=\left[2\Delta \theta \left(\tan_{\tau }\left(\theta \xi \right)\right)\right]e^{\iota \eta },$$

(4.26) $$\chi_{i,22}(r,t)=-\left[2\Delta \theta \left(\cot_{\tau }\left(\theta \xi \right)\right)\right]e^{\iota \eta },$$

(4.27) $$\chi_{i,23}(r,t)=\left[2\Delta \theta \left(\tan_{\tau }\left(2\theta \xi \right)\pm \sqrt{\delta \sigma }\sec_{\tau }\left(2\theta \xi \right)\right)\right]e^{\iota \eta },$$

(4.28) $$\chi_{i,24}(r,t)=\left[2\Delta \theta \left(-\cot_{\tau }\left(2\theta \xi \right)\pm \sqrt{\delta \sigma }\csc_{\tau }\left(2\theta \xi \right)\right)\right]e^{\iota \eta }$$

and

(4.29) $$\chi_{i,25}(r,t)=\left[\Delta \theta \left(\tan_{\tau }\left(\frac{\theta }{2}\xi \right)-\cot_{\tau }\left(\frac{\theta }{2}\xi \right)\right)\right]e^{\iota \eta }.$$

**(6)** For ϕ=0 and θ=−ψ, we have

(4.30) $$\chi_{i,26}(r,t)=\left[2\Delta \theta \left(\tanh_{\tau }\left(\theta \xi \right)\right)\right]e^{\iota \eta },$$

(4.31) $$\chi_{i,27}(r,t)=\left[2\Delta \theta \left(\coth_{\tau }\left(\theta \xi \right)\right)\right]e^{\iota \eta },$$

(4.32) $$\chi_{i,28}(r,t)=-\left[2\Delta \theta \left(-\tanh_{\tau }\left(2\theta \xi \right)\pm \iota {\sqrt{\delta \sigma }}_{\tau }\left(2\theta \xi \right)\right)\right]e^{\iota \eta },$$

(4.33) $$\chi_{i,29}(r,t)=-\left[2\Delta \theta \left(-\coth_{\tau }\left(2\theta \xi \right)\pm {\sqrt{\delta \sigma }}_{\tau }\left(2\theta \xi \right)\right)\right]e^{\iota \eta }$$

and

(4.34) $$\chi_{i,30}(r,t)=\left[\Delta \theta \left(\tanh_{\tau }\left(\frac{\theta }{2}\xi \right)+\coth_{\tau }\left(\frac{\theta }{2}\xi \right)\right)\right]e^{\iota \eta }.$$

**(7)** For $\phi^{2}=4\theta \psi$, we have

(4.35) $$\chi_{i,31}(r,t)=\left[\frac{-2\Delta }{\xi \ln(\tau )}\right]e^{\iota \eta }.$$

**(8)** For ϕ=x,θ=xy(y≠0) and ψ=0, we have

(4.36) $$\chi_{i,32}(r,t)=\left(\Delta x\right)e^{\iota \eta }.$$

**(9)** For ϕ=ψ=0, we have

(4.37) $$\chi_{i,33}(r,t)=0.$$

**(10)** For ν=ϕ=0, we have

(4.38) $$\chi_{i,34}(r,t)=-\left[\frac{2\Delta }{\xi \ln(\tau )}\right]e^{\iota \eta }.$$

(11) For ϕ≠0 and θ=0, we have

(4.39) $$\chi_{i,35}(r,t)=\pm \Delta \phi \left[1-\frac{2\delta }{\cosh_{\tau }\left(\phi \xi \right)-\sinh_{\tau }\left(\phi \xi \right)+\delta }\right]e^{\iota \eta }$$

and

(4.40) $$\chi_{i,36}(r,t)=\pm \Delta \phi \left[1-2\left(\frac{\cosh_{\tau }\left(\phi \xi \right)+\sinh_{\tau }\left(\phi \xi \right)}{\cosh_{\tau }\left(\phi \xi \right)+\sinh_{\tau }\left(\phi \xi \right)+\sigma }\right)\right]e^{\iota \eta }.$$

**(12)** For ϕ=x,ψ=xy(y≠0) and θ=0, we have

(4.41) $$\chi_{i,37}(r,t)=\Delta x\left[1-\frac{2\delta y\tau^{x\xi }}{\delta -\sigma y\tau^{x\xi }}\right]e^{\iota \eta }.$$

## 5 Graphical description

**Figure 1:**
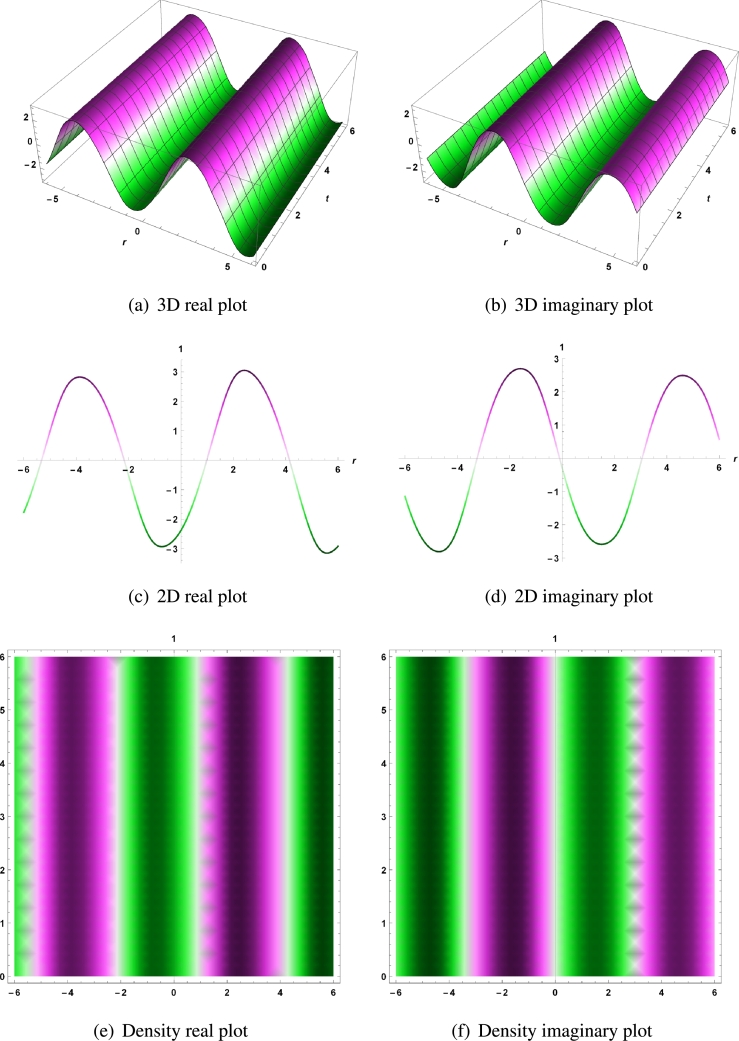
For real and imaginary part of solution *χ*_1,3_, 3D, 2D and density plots with parameters *τ* = 2,*δ* = 0.07,*σ* = 0.5,*ψ* = 3,*θ* = 2,*ϕ* = 4,*a* = 0.5,*b* = −7.75,*c* = −3.5,*μ*_1_ = −2.5,*ν*_1_ = 2,*f*_1_ = 1,*ω*_1_ = 0.5,*d*_1_ = 1.5,*e*_1_ = −1, and for *t* = 1.

In this section the final results are graphically illustrated by using the 2D, 3D and density graphs, to understand the dynamical behaviour of coupled Radhakrishnan-Kundu-Lakshmanan equation. The solutions are visualized as real and imaginary parts separately because these answers are included in the category of complex numbers. Since these solutions involve a variety of arbitrary constants, the graphical representation of these solutions highlights the rich physical phenomena and localized waves of RKLE for the appropriate choice of the involved constants. The discovered solutions are hyperbolic, periodic, trigonometric, bright and dark, combined bright-dark, and W-shaped soliton. These solutions have some physical significance. For example, a dark soliton has lower intensity than background. It isn't produced by a conventional pulse and essentially have no energy in a continuous time beam. Also periodic wave refers to a wave whose wavelength and frequency are determined by a repeating continuous pattern. The real and imaginary parts for Equation (2.7) are represented by the 3D, 2D and density plots with parameters $\tau =2,\delta =0.07,\sigma =0.5,\psi =3,\theta =2,\phi =4,a=0.5,b=-7.75,c=-3.5,\mu_{1}=-2.5,\nu_{1}=2,f_{1}=1,\omega_{1}=0.5,d_{1}=1.5,e_{1}=-1$, and for t=1 in Fig. 1 (a - f).

**Figure 2:**
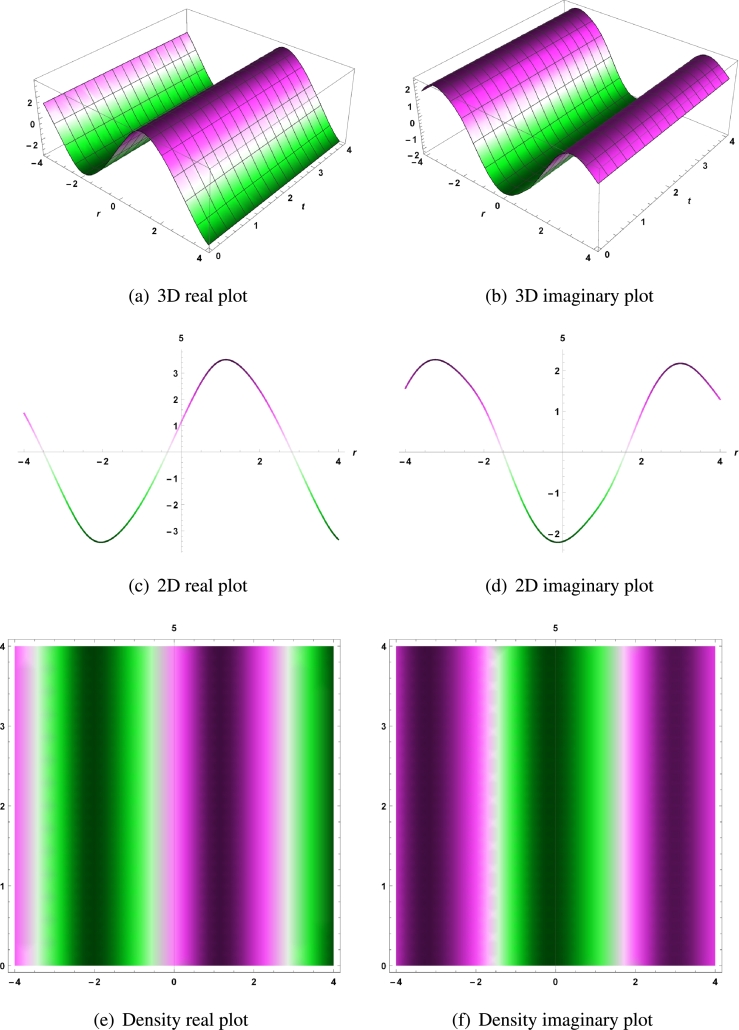
For real and imaginary part of solution *χ*_1,3_, 3D, 2D and density plots with parameters *τ* = 2,*δ* = 0.07,*σ* = 0.5,*ψ* = 3,*θ* = 2,*ϕ* = 4,*a* = 0.5,*b* = −7.75,*c* = −3.5,*μ*_1_ = −2.5,*ν*_1_ = 2,*f*_1_ = 1,*ω*_1_ = 0.5,*d*_1_ = 1.5,*e*_1_ = −1, and for *t* = 5.

The real and imaginary parts for Equation (2.7) are represented by the 3D, 2D and density plots with parameters $\tau =2,\delta =0.07,\sigma =0.5,\psi =3,\theta =2,\phi =4,a=0.5,b=-7.75,c=-3.5,\mu_{1}=-2.5,\nu_{1}=2,f_{1}=1,\omega_{1}=0.5,d_{1}=1.5,e_{1}=-1$, and for t=5 in Fig. 2 (a - d).

**Figure 3:**
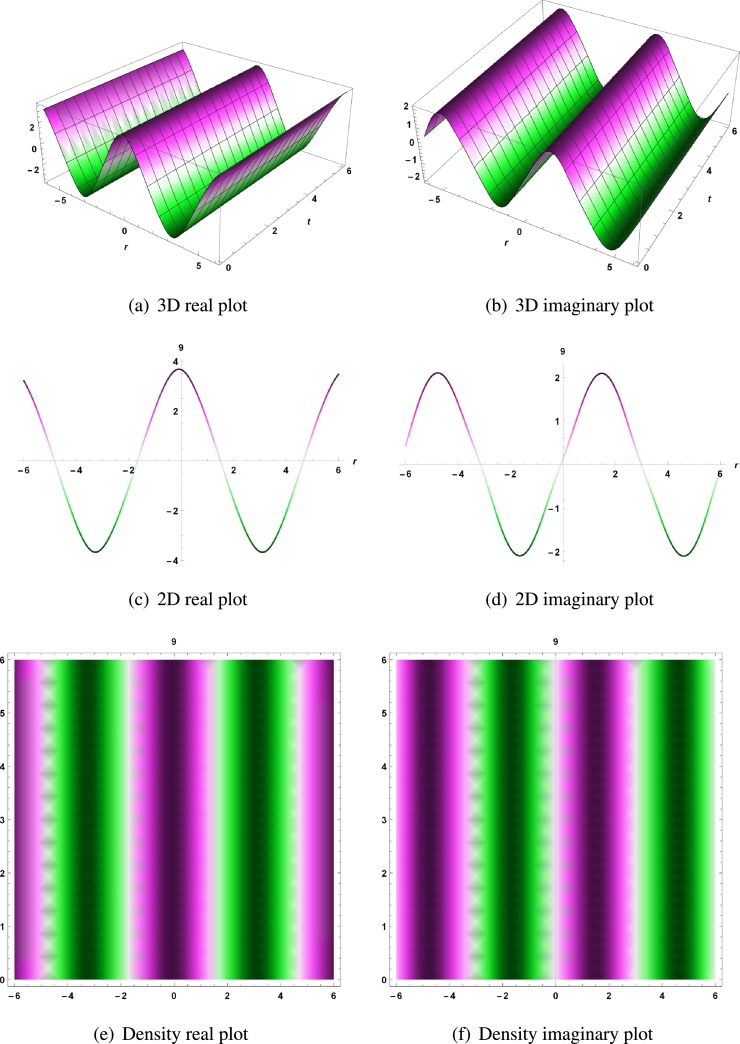
For real and imaginary part of solution *χ*_1,3_, 3D,2D and density plots with parameters *τ* = 2,*δ* = 0.07,*σ* = 0.5,*ψ* = 3,*θ* = 2,*ϕ* = 4,*a* = 0.5,*b* = −7.75,*c* = −3.5,*μ*_1_ = −2.5,*ν*_1_ = 2,*f*_1_ = 1,*ω*_1_ = 0.5,*d*_1_ = 1.5,*e*_1_ = −1, and for *t* = 9.

The real and imaginary parts for Equation (2.7) are represented by the 3D, 2D and density plots with parameters $\tau =2,\delta =0.07,\sigma =0.5,\psi =3,\theta =2,\phi =4,a=0.5,b=-7.75,c=-3.5,\mu_{1}=-2.5,\nu_{1}=2,f_{1}=1,\omega_{1}=0.5,d_{1}=1.5,e_{1}=-1$, and for t=9 in Fig. 3 (a - d).

**Figure 4:**
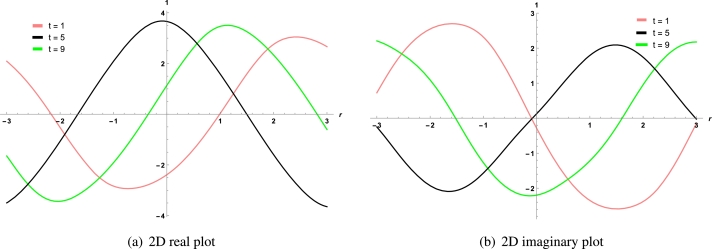
For real and imaginary part of solution *χ*_1,3_, 2D plots for different values of *t*.

Finally the 2D visualization of real and imaginary solutions for three different values of *t* is given in Fig. 4 (a, b), which represents the effect of time on the shape of soliton.

## 6 Conclusions

In this article, we implement the extended direct algebraic approach to extract the novel soliton solutions, to the Radhakrishnan-Kundu Lakshmanan equation (RKLE). The resulting solutions for travelling waves have a single velocity and they assist in improving the efficiency of transmission networks used in the telecommunications industry. They have a considerable impact on optical fibre as well. The obtained results are hyperbolic, periodic, trigonometric, bright and dark, combined bright-dark and W-shaped soliton. To show the physical behaviour of solutions certain results are shown in 2D, 3D and density graphs by selecting the suitable choices of the parameters. The purpose of this work is to locate new, precisely determined solitons for the Radhakrishnan Kundu Lakshmanan model that have never been found before. The computational work and simplicity of the extracted solutions show that the applied methodology is concise, direct, and effective and may be useful in many domains, such as telecommunication engineering, mathematical biology, mathematical physics, and optical fibre. In future it may also be applied to more complicated phenomena with the use of symbolic computation to obtain a range of solitons using a single approach.

## CRediT authorship contribution statement

**Ayesha Mahmood:** Formal analysis, Investigation, Writing – original draft. **Hari Mohan Srivastava:** Conceptualization, Investigation, Project administration. **Muhammad Abbas:** Conceptualization, Investigation, Project administration. **Farah Aini Abdullah:** Formal analysis, Investigation, Writing – review & editing. **Pshtiwan Othman Mohammed:** Conceptualization, Data curation, Funding acquisition, Supervision, Writing – review & editing. **Dumitru Baleanu:** Conceptualization, Funding acquisition, Investigation. **Nejmeddine Chorfi:** Investigation, Validation, Visualization, Writing – review & editing.

## Declaration of Competing Interest

The authors declare that they have no known competing financial interests or personal relationships that could have appeared to influence the work reported in this paper.

## Data Availability

No data was used for the research described in the article.
